# An IL-15 Superagonist, ALT-803, Enhances Antibody-Dependent Cell-Mediated Cytotoxicity Elicited by the Monoclonal Antibody NEO-201 Against Human Carcinoma Cells

**DOI:** 10.1089/cbr.2018.2628

**Published:** 2019-04-10

**Authors:** Massimo Fantini, Justin M. David, Hing C. Wong, Christina M. Annunziata, Philip M. Arlen, Kwong Y. Tsang

**Affiliations:** ^1^ Precision Biologics, Inc., Rockville, Maryland.; ^2^ Altor BioScience Corporation, Miramar, Florida.; ^3^ Women's Malignancy Branch, Center for Cancer Research, National Cancer Institute, National Institutes of Health, Bethesda, Maryland.

**Keywords:** antibody-dependent cell-mediated cytotoxicity, IL-15 superagonist, immunostimulation, monoclonal antibody, NEO-201, natural killer cells

## Abstract

***Background:*** A major mechanism of action for therapeutic antibodies is antibody-dependent cell-mediated cytotoxicity (ADCC). ALT-803 is an interleukin-15 superagonist complex that enhances ADCC against human carcinoma cells *in vitro* and exerts an antitumor activity in murine, rat, and human carcinomas *in vivo*. The authors investigated the ability of ALT-803 to modulate ADCC mediated by the humanized IgG1 monoclonal antibody (mAb) NEO-201 against human carcinoma cells.

***Materials and Methods:*** ALT-803 modulating activity on ADCC mediated by NEO-201 was evaluated on several NEO-201 ligand-expressing human carcinoma cells. Purified human natural killer (NK) cells from multiple healthy donors were treated with ALT-803 before their use as effectors in ADCC assay. Modulation of NK cell phenotype and cytotoxic function by exposure to ALT-803 was evaluated by flow cytometry and gene expression analysis.

***Results:*** ALT-803 significantly enhanced ADCC mediated by NEO-201. ALT-803 also upregulated NK activating receptors, antiapoptotic factors, and factors involved in the NK cytotoxicity, as well as downregulated gene expression of NK inhibiting receptors.

***Conclusions:*** These findings indicate that ALT-803 can enhance ADCC activity mediated by NEO-201, by modulating NK activation and cytotoxicity, suggesting a possible clinical use of ALT-803 in combination with NEO-201 for the treatment of human carcinomas.

## Introduction

Conventional cancer therapies, such as surgery, radiation, and chemotherapy, are usually effective in treating patients with the primary tumors, but often fail in treating metastatic disease.^1,2^ The development of immunotherapies for cancer has led to prolonged clinical responses in metastatic disease, even when traditional therapies have failed.^3^ The immune system suppresses the proliferation of cancer cells, recognizing cancer-specific antigens, and activating various cytotoxic programs, through a process called immunoediting of the cancer cells.^4^ However, cancer cells may escape the selective pressure of the immune system, generating a more malignant phenotype with the ability to spread to different parts from the primary site (in a process known as metastasis).^5^ The aim of cancer immunotherapy is to enhance antitumor immune responses to control the growth of both the primary tumor and cancer cells present in the blood circulation and metastases in distant organs.^6–10^

Cellular subsets within the innate immune system, including natural killer (NK) cells, play a significant role in cancer immunosurveillance, and monoclonal antibodies (mAbs) in combination with immunostimulants can enhance innate antitumor immunity.^10,11^ Several studies have shown the antitumor potential of NK cells, demonstrating the potential value of NK cell-based immunotherapies as a new frontier in cancer treatment.^12–14^

NK cell antitumor activity can be modulated by the use of cytokines. The cytokine interleukin-15 (IL-15) plays a crucial role in the immune system by affecting NK cell development, proliferation, cytotoxicity, and cytokine production.^15^ IL-15 binds to the IL-15Rα present on the surface of monocytes or dendritic cells, and is presented to NK and CD8^+^ T cells where it forms a complex with IL-15Rβ to activate several intracellular signaling pathways, such as the PI3K–AKT–mTOR pathway.^16,17^

Technologies based on IL-15's immunomodulatory properties have also been developed, including the IL-15 superagonist complex (ALT-803). ALT-803 consists of an IL-15 variant (IL-15N72D) bound to an IL-15 receptor α/IgG1 Fc fusion protein, resulting in improved stability, longer persistence in lymphoid tissues, and enhanced antitumor activity compared to native IL-15 *in vivo*.^18^

ALT-803 was found to enhance antibody-dependent cell-mediated cytotoxicity (ADCC) against a wide range of human carcinoma cells *in vitro*.^19–21^ ALT-803 also exerted a significant antitumor activity in murine models of multiple myeloma, glioblastoma, ovarian cancer, and in murine breast, colon, and melanoma tumor-bearing mice.^22–26^ Moreover, intravesical ALT-803 and Bacillus Calmette-Guerin (BCG) administration showed a reduction of tumor burden in a bladder cancer rat model.^27^ Promising preclinical data on the use of ALT-803 as an anticancer drug have spurred interest in using it in the clinic. Several ongoing clinical trials are evaluating the safety and efficacy of ALT-803 alone or in combination with conventional cancer treatments.^28–35^

NEO-201 is a humanized IgG1 mAb that reacts to tumor-associated antigens derived from the Hollinshead allogeneic colorectal cancer vaccine platform.^36–38^ A preliminary study indicated that NEO-201 may recognize tumor-associated variants of CEACAM family members.^39^ In a previous study, the authors showed that NEO-201 is remarkably tumor specific in its staining profile and demonstrated its ability to react to a wide range of human carcinoma cell lines by flow cytometry and tumor tissues by immunohistochemistry. It has also been shown that an overwhelming majority of healthy normal tissues were found to be negative for NEO-201.^40^Although NEO-201 positivity was detected in normal tongue and ectocervix tissues, the staining intensity was weak.^39,40^ NEO-201 administration did not induce any grossly observable toxicity in mice. A single-dose toxicity study in nonhuman primates demonstrated safety and tolerability of NEO-201, as a transient decrease in circulating neutrophils was the only related adverse effect observed.^40^

In addition, NEO-201 exhibited both ADCC and complement-dependent cytotoxicity (CDC) activity against human carcinoma cells *in vitro*, and counteracted the growth of human pancreatic xenograft tumors *in vivo*.^40^

ADCC is mediated by binding of the constant region (fragment crystallizable, Fc) of mAbs and the Fc gamma receptor IIIa (FcƴRIIIa, CD16) expressed on macrophages and NK cells. This interaction induces macrophages to phagocytose mAb-opsonized cancer cells, and leads NK cells to lyse mAb-bounded cancer cells.^41^

This study was undertaken to assess the ability of ALT-803 to enhance the ADCC mediated by NEO-201, employing NK cells treated with ALT-803 as effectors against human carcinoma cells. The authors demonstrated that ALT-803 significantly enhanced the ADCC activity mediated by NEO-201 against NEO-201-positive carcinoma cells in a dose-dependent manner. ALT-803 was also able to upregulate gene and protein expression of NK-activating receptors and the gene expression of antiapoptotic factors and factors involved in NK cytotoxicity, as well as to downregulate gene expression of NK-inhibiting receptors and factors involved in NK cell exhaustion, and to prolong NK cell viability.

These findings provide the rationale for the development of a potential clinical therapy using ALT-803 in combination with NEO-201 as a strategy for the treatment of NEO-201-positive tumors.

## Materials and Methods

### Cell lines and culture

The following human carcinoma cell lines were obtained from the American Type Culture Collection (Manassas, VA): pancreas (ASPC-1 and CFPAC-1), breast (ZR-75-1), and lung (H520 and HCC827). All cell cultures were maintained in RPMI 1640 or IMDM culture medium (Corning, Corning, NY) as designated by the provider for propagation and maintenance. Culture medium was supplemented with 10% USA-sourced and heat-inactivated HyClone fetal bovine serum (FBS) defined (GE Healthcare Life Sciences, Issaquah, WA), 100 U/mL penicillin, and 100 μg/mL streptomycin (Corning Life Science, Manassas, VA). Peripheral blood mononuclear cells (PBMCs) from healthy volunteer donors were obtained from the National Institutes of Health Clinical Center Blood Bank (NCT00001846) under the appropriate Institutional Review Board approval and informed consent.

### NK cell purification

NK effector cells were isolated from PBMCs using the EasySep Human NK Cell Isolation Kit (StemCell Technologies, Vancouver, BC, Canada) according to the manufacturer's protocol. NK cells isolated from healthy donors were treated for 24 and 48 h with ALT-803 (Altor BioScience, Miramar, FL) at different concentrations (6.25, 12.5, and 25 ng/mL) before being examined by flow cytometry, by NanoString Technologies nCounter Platform, or to be used as effectors in the ADCC assay. Untreated NK cells were used as control.

### Flow cytometry

Analysis of the expression of cell surface and intracellular proteins in purified NK cells and in human carcinoma cell lines was performed by flow cytometry. Cells (1.0 × 10^6^) were incubated with 1 μL per test of LIVE/DEAD Fixable Aqua (Thermo Fisher Scientific, Waltham, MA) in 1 × phosphate buffered saline (PBS) for 30 min at 4°C to accomplish live versus dead cell discrimination. Cells were then centrifuged, washed twice with cold PBS, and then stained with primary antihuman mAbs in 1 × PBS +1% BSA (Teknova, Hollister, CA) for 30 min at 4°C. Binding of NEO-201 to human carcinoma cell lines was detected by Pacific Blue-conjugated NEO-201 antibody (BioLegend, San Diego, CA). To detect the NK markers modulated by ALT-803, purified NK cells were labeled with following antibodies: CD56-PE (clone 5.1H11), CD16-PerCP-Cy5.5 (clone 3G8), Tim-3-PE-Cy7 (clone F38–2E2), NKG2D-BV421 (clone 1D11), CD107a-APC-Cy7 (clone H4A3), Granzyme B-FITC (clone GB11), PD-1-APC (clone EH12.2H7), and CD158d-APC (clone mAb 33) (BioLegend). After staining, cells were washed twice with cold PBS and examined using a FACSVerse flow cytometer (BD Biosciences, San Jose, CA). Analysis of cellular fluorescence was performed using BD FACSuite software (BD Biosciences). Positivity was determined by using fluorescence-minus-one controls.

### ADCC assay

Purified NK cells were treated with ALT-803 (6.25, 12.5, and 25 ng/mL) or vehicle control (RPMI-1640 medium supplemented with l-glutamine, 10% FBS, and antibiotics) for 48 h before being used as effectors. On the day of the assay, human carcinoma cell lines (target cells) were labeled with 10 μM Calcein AM cell-permeant dye (Thermo Fisher Scientific) for 30 min and then seeded in triplicate at 3.0 × 10^3^ cells/well into black-walled flat-bottom 96-well culture plates (655090; Greiner Bio-One, Kremsmünster, Austria). Target cells were then treated with human IgG1 isotype control antibody (Thermo Fisher Scientific) or NEO-201 (dose range 0.1–10 μg/mL), and then, purified NK cells were added at effector-to-target (E:T) ratios of 6.25:1 and 12.5:1.

For blocking studies, purified NK cells were incubated at 37°C for 2 h with 15 μg/mL of antihuman CD16-neutralizing mAb (eBioscience, San Diego, CA) before being used as effectors. After 4 h of incubation at 37°C, 10 μg/mL the propidium iodide (Thermo Fisher Scientific) was added to each well and the plate was imaged and analyzed using the Celigo Imaging Cytometer (Nexcelom Bioscience LLC, Lawrence, MA). Specific ADCC lysis was calculated using the following formula: % specific lysis = 100 − [(average live target count experimental/average live target count control) × 100].

### Gene expression analysis

The gene expression analysis of ALT-803-treated NK cells was performed using the NanoString Technologies nCounter Platform. Briefly, purified NK cells were exposed to vehicle control (RPMI-1640 medium supplemented with l-glutamine, 10% FBS, and antibiotics) or ALT-803 (6.25, 12.5, and 25 ng/mL) for 48 h before performing RNA isolation. Total RNA was purified using the RNeasy Plus Mini Kit (Qiagen, Valencia, CA). RNA extraction and purification steps were performed according to the manufacturers' instructions. RNA concentration and purity were determined using Nanodrop 2000 (Thermo Fisher Scientific). RNA was then analyzed for gene expression using the Nanostring Technologies nCounter LBL-C0269 Human Immunology v2 Panel (Nanostring Technologies, Inc., Seattle, WA).

### Analysis of cell viability

To analyze the effect of ALT-803 on NK cell viability, purified NK cells from two healthy donors were exposed to vehicle control or ALT-803 (25 ng/mL) on day 0 and then returned to incubation at 37°C in presence of 5% CO_2_ for 6 d. Alternatively, vehicle control and ALT-803 were replenished every 2 d.

After drug exposure, cells were harvested and viable cells were counted by AO/PI (acridine orange/propidium iodide) staining solution (Nexcelom Bioscience LLC) using the Cellometer Auto T4 automated cell counter (Nexcelom Bioscience LLC).

### Statistical analysis

The distribution of cell populations by flow cytometry analyses was preliminary verified using the Kolmogorov–Smirnov test.

Significant differences between two treatment groups were determined by *T*-test, and differences between multiple treatment groups were evaluated by two-way ANOVA followed by Turkey's multiple comparison test, using GraphPad Prism 7.0 software (GraphPad Software, La Jolla, CA). Differences were considered significant when the *p*-value was <0.05.

Graphs depict the mean ± SD from one representative experiment performed in triplicate.

## Results

### ALT-803 enhances ADCC mediated by NEO-201 against human carcinoma cells

To investigate the ability of ALT-803 to modulate ADCC activity mediated by NEO-201, ALT-803 was added to human NK cells isolated from PBMCs, from multiple healthy donors in the presence of several NEO-201 ligand-expressing human carcinoma cell lines. The NEO-201-ligand expression profile of human carcinoma cell lines is shown in Table 1.

**Table 1.** Flow Cytometry Analysis of NEO-201 Binding to Cultured Tumor Cell Lines Derived from Various Types of Solid Tumors

*Tumor*	*Cell line*	*% positive*	*MFI*
Pancreas	CFPAC-1	97.70	9276
Squamous lung (NSCLC)	H520	60.51	247
Adenocarcinoma lung (NSCLC)	HCC827	73.39	330
Breast (ER^+^/PR^+^/HER2^+^)	ZR-75-1	51.43	701

The percentage of positive cells and median fluorescence intensity values are detailed for each cell line. NEO-201 positivity was defined as % positive >10%.

MFI, median fluorescence intensity; NSCLC, non-small-cell lung carcinoma.

These NEO-201-positive human carcinoma cell lines were chosen from the panel of human carcinoma cell lines tested for NEO-201 binding reported in the previous study.^40^

NEO-201-negative human carcinoma cell lines were not selected for this investigation.

**FIG. 1.**
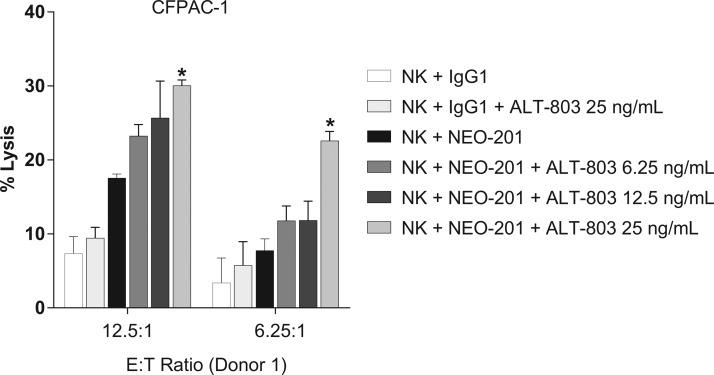
ALT-803 enhances NEO-201-mediated ADCC against CFPAC-1 in a dose-dependent manner. CFPAC-1 (human pancreatic carcinoma cells) were used as target cells. NEO-201 and human IgG1 (negative control) were used at a concentration of 10 μg/mL of NEO-201 in ADCC assay. Purified NK cells from a healthy donor were treated with ALT-803 (6.25–25 ng/mL) or vehicle control for 48 h before being used as effector cells at the indicated E:T ratios. Results are presented as mean ± S.E.M. from three replicate wells. *Asterisks* denote statistical significance of NK+NEO-201+ALT-803 relative to controls (NK+NEO-201; NK+IgG1+ALT-803) (two-way ANOVA). *p < 0.05. ADCC, antibody-dependent cellular cytotoxicity; NK, natural killer.

ALT-803-modulating activity on ADCC mediated by NEO-201 was evaluated first on the highly NEO-201-positive CFPAC-1 cell line. Purified NK cells from one healthy donor were treated with ALT-803 (6.25–25 ng/mL) or vehicle control for 48 h before their use as effectors in the ADCC assay. ALT-803 enhanced NEO-201-mediated ADCC against CFPAC-1 in a dose-dependent manner, compared with the vehicle control at both E:T ratios (Fig. 1). The results demonstrate that ALT-803 significantly increased the ADCC activity mediated by NEO-201 at a concentration as low as 25 ng/mL (Fig. 1). For this reason, the authors chose this concentration (25 ng/mL) for the future experiments.

**FIG. 2.**
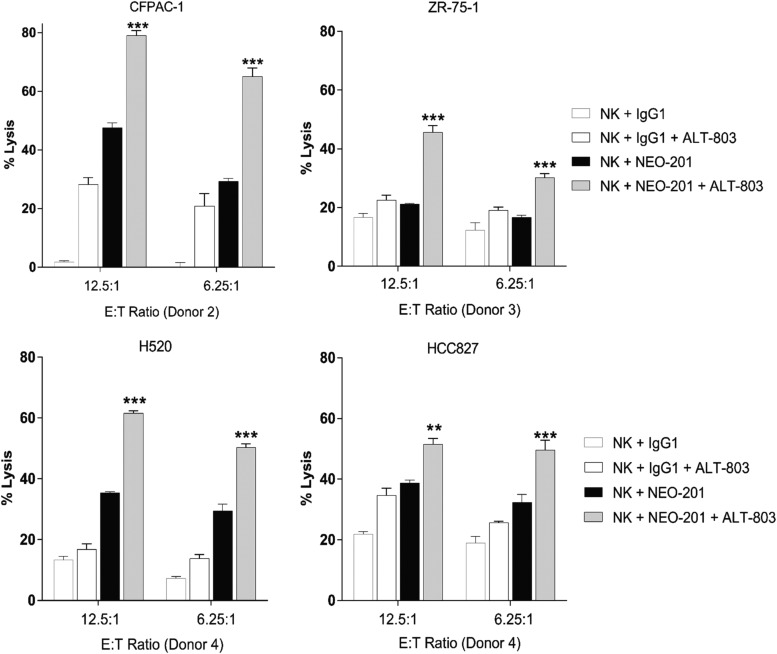
ALT-803 enhances NEO-201-mediated ADCC against human carcinoma cell lines. ALT-803 enhances the ADCC activity mediated by NEO-201 against CFPAC-1, ZR-75-1, H520, and HCC827 cells. Carcinoma cells were used as target cells in the presence of 10 μg/mL of NEO-201 or human IgG1 (negative control) in the ADCC assay. Purified NK cells from three healthy donors were treated with ALT-803 (25 ng/mL) or vehicle control for 48 h before being used as effector cells at the indicated E:T ratios. Results are presented as mean ± S.E.M. from three replicate wells. *Asterisks* denote statistical significance of NK+NEO-201+ALT-803 relative to controls (NK+NEO-201; NK+IgG1+ALT-803) (two-way ANOVA). **p < 0.01; ***p < 0.001. ADCC, antibody-dependent cellular cytotoxicity; NK, natural killer.

To determine whether ALT-803 can modulate the ADCC activity mediated by NEO-201 against other NEO-201-positive cancer cells, ADCC assays were performed using four human carcinoma cell lines: pancreas (CFPAC-1), breast (ZR-75-1), and lung (H520 and HCC827). Purified NK cells from additional healthy donors were treated with ALT-803 (25 ng/mL) or vehicle control for 48 h before being used as effectors in the ADCC assay. As shown in Figure 2, ALT-803 significantly enhanced NEO-201-mediated ADCC at both E:T ratios in all cell lines compared to untreated cells: CFPAC-1 (E:T 12.5:1, 1.66-fold increase; and E:T 6.25:1, 2.22-fold increase), ZR-75 (E:T 12.5:1, 2.15-fold increase; and E:T 6.25:1, 1.80-fold increase), H520 (E:T 12.5:1, 1.74-fold increase; and E:T 6.25:1, 1.71-fold increase), and HCC827 (E:T 12.5:1, 1.33-fold increase; and E:T 6.25:1, 1.53-fold increase). These data demonstrated that ALT-803 treatment of NK cells effectively enhances NEO-201-mediated ADCC against NE0-201-positive carcinoma cells *in vitro*.

### Treatment of NK cells with ALT-803 enhances ADCC against human carcinoma cell lines at suboptimal doses of NEO-201

**FIG. 3.**
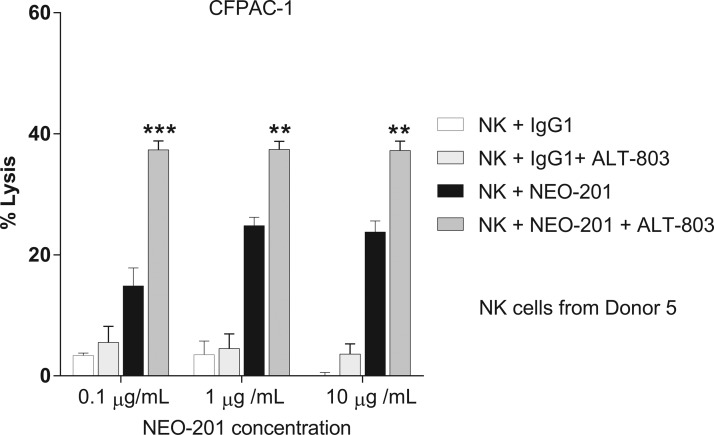
Treatment of NK cells with ALT-803 enhances ADCC against human carcinoma cell lines at suboptimal doses of NEO-201. ALT-803 enhances ADCC activity mediated by NEO-201 against CFPAC-1 cells. CFPAC-1 cells were used as target cells in the presence of NEO-201 or human IgG1 (negative control) at different concentrations (0.1–10 μg/mL) in ADCC assay. Purified NK cells from a healthy donor were treated with ALT-803 (25 ng/mL) or vehicle control for 48 h before being used as effector cells at an E:T ratio of 12.5:1. Results are presented as mean ± S.E.M. from three replicate wells. *Asterisks* denote statistical significance of NK+NEO-201+ALT-803 relative to NK+NEO-201 (two-way ANOVA). **p < 0.01; ***p < 0.001. ADCC, antibody-dependent cellular cytotoxicity; NK, natural killer.

To evaluate whether ALT-803 enhances NEO-201-mediated ADCC at lower antibody doses, the authors performed a NEO-201 antibody titration (0.1, 1, and 10 μg/mL) in the ADCC assay, using CFPAC-1 cells as target. As shown in Figure 3, titration assays revealed that NK cells treated with ALT-803 (25 ng/mL) can enhance NEO-201-mediated ADCC at doses as low as 0.1 μg/mL compared to untreated NK cells. These data suggest that treatment of NK cells with ALT-803 could decrease the dose of NEO-201 needed to achieve its clinical efficacy if used in combination therapy.

### ADCC mediated by NEO-201 enhanced by treatment of NK cells with ALT-803 is dependent on CD16 engagement

**FIG. 4.**
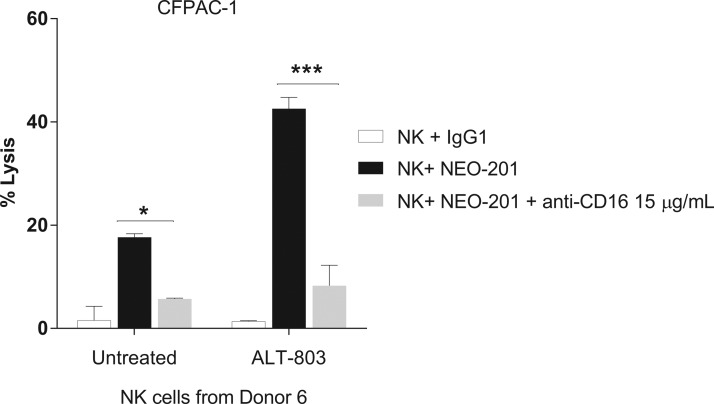
ADCC mediated by NEO-201 can be blocked by the anti-CD16 antibody. CFPAC-1 cells were used as target cells in the presence of 10 μg/mL of NEO-201 or human IgG1 (negative control) in the ADCC assay. Purified NK cells from a healthy donor were treated with ALT-803 (25 ng/mL) or vehicle control for 48 h before being used as effector cells at the E:T ratio of 12.5:1. Where applicable, NK cells were pretreated for 2 h with anti-CD16 blocking antibody (15 μg/mL) before being used as effectors. Results are presented as mean ± S.E.M. from three replicate wells. *Asterisks* denote statistical significance of NK+NEO-201 relative to NK+NEO-201+anti-CD16 in both untreated and treated NK cells (two-way ANOVA). *p < 0.05; ***p < 0.001. ADCC, antibody-dependent cellular cytotoxicity; NK, natural killer.

CD16 engagement has been proven to play a critical role in ADCC mediated by several mAbs clinically approved to treat cancer.^41–47^ To determine whether tumor cell lysis in the presence of NEO-201 is mediated by ADCC and that ALT-803 is able to enhance NEO-201-mediated ADCC, NK cells isolated from a healthy donor were treated with ALT-803 (25 ng/mL) or vehicle control for 48 h. Then, the anti-CD16 antibody was added to NK cells before using them in the ADCC assay. As shown in Figure 4, the anti-CD16 antibody significantly decreased ADCC activity mediated by NEO-201 of both untreated (*p* < 0.05) and ALT-803-treated NK cells (*p* < 0.001), confirming that increased tumor cell lysis in presence of NEO-201 is mediated by ADCC and that ALT-803 enhances specifically NEO-201-mediated ADCC.

### ALT-803 modulates the phenotype of human healthy donor NK cells toward a more active cytotoxic function

Mounting evidence suggests that NK cells exhibit potent antitumor responses following IL-15 priming in humans^15–17,24,48,49^ and in animals.^16,25^ Thus, the authors sought to evaluate whether NK cell phenotype and cytotoxic function could be modulated by exposure to ALT-803. Human NK cells isolated from two healthy donors were treated with ALT-803 (25 ng/mL) or vehicle control for 48 h before performing phenotypical analysis. NK cells treated with ALT-803 showed an increase in TIM-3^+^/NKG2D^+^ expression of 1.69-fold (TIM-3 median fluorescence intensity [MFI] 3.76-fold; and NKG2D MFI 1.63-fold) in Donor 7 and 3.43-fold (TIM-3 MFI 2.95-fold; and NKG2D MFI 2.32-fold) in Donor 8 in the CD16^+^/CD56^+^ population, compared to untreated cells (Table 2).

**Table 2.** Effect of ALT-803 on the Natural Killer Cell Phenotype

% positive (MFI) (Donor 7)									
Treatment	CD16^−^/CD56^+^	CD16^+^/CD56^+^	^*^TIM-3^+^	^*^NKG2D^+^	^*^TIM-3^+^/NKG2D^+^	^*^CD107a^+^	^*^Granzyme B^+^	^*^PD-1^+^	^*^CD158d^+^
Untreated	9.51 (52/3457)	89.27 (7434/1048)	42.87 (843)	87.99 (317)	43.95 (799/307)	99.99 (887)	98.84 (474)	12.82 (907)	14.58 (167)
ALT-803	9.03 (84/**15,419**)	90.97 (6,915/**3605**)	**81.56** (**2972**)	78.33 (**590**)	**74.68** (**3011**/**500**)	99.91 (**2711**)	99.88 (**1346**)	16.18 (1272)	17.68 (**1064**)

% positive (MFI) (Donor 8)									
Treatment	CD16^−^/CD56^+^	CD16^+^/CD56^+^	^*^TIM-3^+^	^*^NKG2D^+^	^*^TIM-3^+^/NKG2D^+^	^*^CD107a^+^	^*^Granzyme B^+^	^*^PD-1^+^	^*^CD158d^+^
Untreated	12.27 (83/908)	87.73 (6425/3043)	33.92 (827)	79.46 (465)	24.85 (853/511)	99.95 (13,775)	99.75 (1157)	5.36 (211)	12.72 (227)
ALT-803	13.23 (91/757)	86.77 (5848/**7810**)	**92.14 (2462)**	**95.40 (1152)**	**85.30 (2517/1190)**	100 (10,365)	100 (**2457**)	3.87 (229)	11.00 (219)

NK cells were treated with ALT-803 (25 ng/mL) or vehicle control (untreated) for 48 h, before being analyzed by flow cytometry. Data are presented as percentage of viable cells expressing the cell surface proteins relative to untreated cells. Values in brackets represent MFI for each NK marker. Values in bold represent an increase equal to or above 40% in protein levels and/or MFI in treated cells compared to untreated cells.

^*^ Percent of the specific NK marker in the CD16^+^/CD56^+^ population.

MFI, median fluorescence intensity; NK, natural killer.

ALT-803 also induced a marked increase of CD56 and granzyme B MFI in CD16^+^/CD56^+^ population in both donors (Donor 7: CD56, 3.43-fold; granzyme B, 2.84-fold. Donor 8: CD56, 2.57-fold; granzyme B, 2.12-fold) and of CD107a MFI (3.05-fold) in the CD16^+^/CD56^+^ population in one donor, compared to untreated cells (Table 2).

### ALT-803 modulates the gene expression of human healthy donor NK cells

To better understand the mechanism whereby ALT-803 modulates NK function, the authors performed a gene expression analysis on NK cells treated with ALT-803. Purified NK cells from a healthy donor were treated with ALT-803 at different concentrations (6.25–25 ng/mL) or with vehicle control for 48 h before performing RNA isolation. RNA from the ALT-803 cultured cells was analyzed for gene expression using Nanostring Technologies nCounter LBL-C0269 Human Immunology v2 Panel. As shown in Table 3, ALT-803 upregulated gene expression of NK-activating receptors (LAG-3 and 4–1BB) and downregulated gene expression of NK inhibitory receptors (KLRG1 and CD85C).

**Table 3.** Modulation of Categorized Transcripts Expressed in Human Natural Killer After Treatment with ALT-803

*Activating receptors*					
*Gene name*	*Function*	*Refs.*	*ALT-803 25 ng/mL*	*ALT-803 12.5 ng/mL*	*ALT-803 6.25 ng/mL*
LAG3 (lymphocyte activation gene 3) or CD223	Expressed on activated NK cells. Encodes for a receptor for MHC class II molecules.	^50^	0.95	0.96	**1.9**
TNFRSF9 (TNF receptor superfamily member 9) or CD137 or 4-1BB	Expressed on activated human NK cells. It is associated to the release of proinflammatory cytokines	^51^	**2.53**	**2.64**	**2.52**

*Inhibitory receptors*					
*Gene name*	*Function*	*Refs.*	*ALT-803 25 ng/mL*	*ALT-803 12.5 ng/mL*	*ALT-803 6.25 ng/mL*
KLRG1 (killer cell lectin-like receptor G1)	Impairs the capacity of NK cells to proliferate and to produce IFN-γ, and it is positively associated with apoptosis of NK cells in response to inflammatory cytokine stimulation.	^52^	−1.34	−1.32	−**1.95**
LILRB5 (leukocyte immunoglobulin-like receptor B5) or CD85C	Involved in the negative regulation of immune cell activation	^53^	−0.2	−**1.77**	−0.63

*Factors involved in the NK cytotoxicity*					
*Gene name*	*Function*	*Refs.*	*ALT-803 25 ng/mL*	*ALT-803 12.5 ng/mL*	*ALT-803 6.25 ng/mL*
CLU (clusterin)	Regulates the expansion and the effector function of NK cells. Clusterin synergizes with IL-2 to enhance IFN-γ production by NK cells	^54^	**2.04**	−0.12	0.95
GBP1 (guanylate binding protein 1)	Promotes oxidative killing. GBP1 upregulation was correlated with better prognosis in breast cancer patients	^55,56^	**2.04**	**1.71**	**2.02**
GZMA (granzyme A)	Induces apoptosis in target cells	^57^	1.42	1.32	**1.64**
GZMB (granzyme B)	Induces apoptosis in target cells	^57^	**3.19**	**3.29**	**3.38**
IDO1 (indoleamine 2,3-dioxygenase 1)	Plays a role in antitumor immunity by regulating cytotoxic activity of NK cells.	^58^	**1.69**	**2.59**	**2.55**
IFITM1 (interferon induced transmembrane protein 1) or CD225	CD225 expression is upregulated by IFN-γ. It is involved in the physiological process of immune response signaling	^59,60^	1.5	1.57	**1.73**
PRF1 (perforin 1)	Plays a key role in secretory granule-dependent cell death, leading to the lysis of target cells	^61^	**2.13**	**2.03**	**2.12**
NCAM1 (neural cell adhesion molecule 1) or CD56	Archetypal phenotypic marker of mature NK cells. Marker for cytotoxicity in NK cells	^62^	**2.01**	**2.21**	**2.03**

*Cytokines/chemokine and receptors*					
*Gene name*	*Function*	*Refs.*	*ALT-803 25 ng/mL*	*ALT-803 12.5 ng/mL*	*ALT-803 6.25 ng/mL*
CSF2 (colony- stimulating factor 2) (GM-CSF)	Promotes survival and activation of NK cells	^63^	**3.67**	**4.39**	**4.17**
CXCL9 (C-X-C motif chemokine ligand 9)	CXCL9 is upregulated by IFN-γ.	^63^	**3.13**	**2.69**	**4.27**
	Increases immunogenicity of NK cells				
CXCL10 (C-X-C motif chemokine ligand 10)	CXCL10 is upregulated by IFN-γ.	^63^	**5.61**	**5.16**	**7.19**
	Exerts different antitumor effects				
CX3CR1 (chemokine receptor 1) or (fracralkine receptor 1)	IL-15 increases CX3CR1 expression on NK cells. The interaction between CX3CL1 and CXCR1 enhances NK cell adhesion to target cells and NK killing of CX3CL1-expressing targets	^64,65^	**1.64**	1.47	1.42
IFNG (interferon gamma)	TYPE II interferon: contributes to NK cell homeostasis, activation, and antitumor function	^66^	**3.53**	**3.51**	**3.1**
TNF (tumor necrosis factor) or TNF-α	IFN-γ and TNF-α synergistically enhance NK cell cytotoxicity through NF-κB-dependent upregulation of ICAM-1 expression in target cells	^66^	**1.66**	**2.01**	**1.79**
LTA (lymphotoxin α) or TNF-β	Plays a role in the apoptotic killing of tumor cells by human NK cells	^67^	**2.23**	**3.03**	**2.65**
TNFSF8 (TNF superfamily member 8) or CD153 or CD30L	Expressed on activated NK cells. Is involved in the apoptotic killing of tumor cell targets expressing CD30.	^67^	**1.63**	**1.72**	**2.02**
TNFSF10 (TNF superfamily member 10) or CD253 (TRAIL)	Expressed on activated NK cells. TRAIL–TRAIL-receptor interaction induces apoptosis on target cells.	^67,68^	1.2	**1.61**	**1.63**
IL2RA (IL 2 receptor subunit alpha) or CD25	Preactivation with IL-12, IL-15, and IL-18 enhances CD25 expression on NK cells. NK cells expressing high amount of CD25 have higher cytotoxic activity.	^69^	**5.74**	**6.38**	**6.07**
IL2RB (IL 2 receptor subunit beta) or CD122	Confers IL-15 responsiveness and enhances NK cell activation process	^70^	1.47	1.54	**1.63**

*Signal transduction and transcriptional factors*					
*Gene name*	*Function*	*Refs.*	*ALT-803 25 ng/mL*	*ALT-803 12.5 ng/mL*	*ALT-803 6.25 ng/mL*
BCL2 (B cell lymphoma 2)	Bcl-2 is a major antiapoptotic protein. It is expressed in human NK cells and is regulated by IL-2	^71^	**2.91**	**2.74**	**2.92**
DUSP4 (dual specificity protein phosphatase 4) called also MKP-2	Expressed in human NK cells. Protects against genotoxic stress-induced apoptosis.	^72,73^	**1.95**	**2.19**	**2.06**
PDCD1 (programmed cell death 1) or PD-1 or CD279	Plays a critical role in mediating NK cell exhaustion.	^74^	0.52	−**1.92**	0.15
	PD-1^+^ NK cells fail to degranulate and release IFN-γ, but exogenous IL-2 or IL-15 can restore this defect				

NK cells were exposed to ALT-803 (6.25–25 ng/mL) for 48 h, before performing RNA isolation. RNA was then analyzed for gene expression using the Nanostring Technologies nCounter LBL-C0269 Human Immunology v2 Panel. Data are presented as log2-fold change in the gene expression of ALT-803-treated NK cells versus untreated NK cells. Values in bold represent a gene expression change above 1.6 log2-folds.

IL, interleukin; NK, natural killer.

ALT-803 upregulated gene expression of factors involved in NK cytotoxicity (clusterin, IDO1, granzyme A and B, CD225, perforin 1, and CD56), and of cytokines/chemokines and receptors (GM-CSF, CXCL9 and 10, chemokine receptor 1, interferon-γ [IFN-γ], tumor necrosis factor-α [TNF-α], TNF-β, CD30L, TRAIL, CD25, and CD122). ALT-803 also modulated gene expression of signal transduction and transcriptional factors, upregulating the mRNA transcription of the antiapoptotic protein Bcl-2 and DUSP-4, and downregulating the mRNA transcription of factors involved in NK cell exhaustion (PD-1). Altogether, these data indicate that ALT-803 is able to modulate the phenotype of human NK cells, through upregulation of gene and protein expression of NK-activating receptors and gene expression of antiapoptotic factors and factors involved in the NK cytotoxicity, and through the downregulation of gene expression of NK-inhibiting receptors and factors involved in the NK cell exhaustion.

### ALT-803 prolongs viability of NK cells

**FIG. 5.**
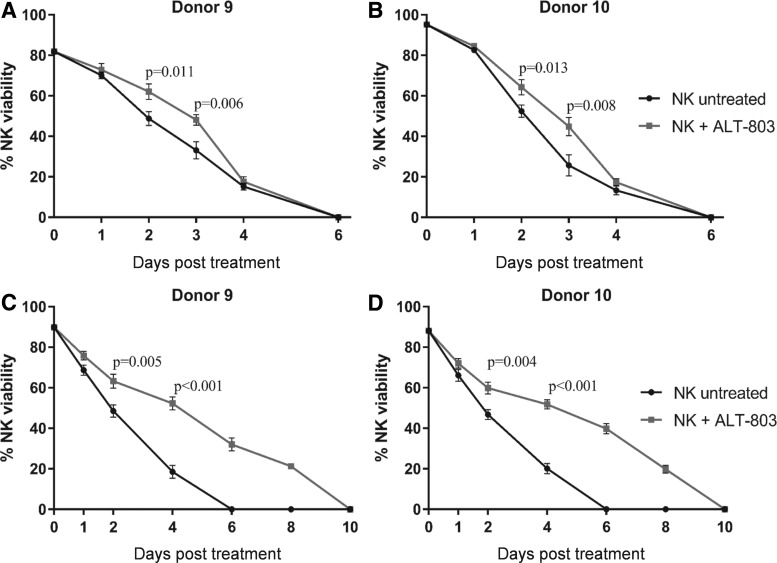
ALT-803 prolongs viability of NK cells. Purified NK cells from two healthy donors were treated with ALT-803 (25 ng/mL) or vehicle control at the day 0 and then returned to incubation for 6 d. NK cell viability was analyzed on day 1, 2, 3, 4, and 6 post-treatment **(A, B)**. Purified NK cells from two healthy donors were treated with ALT-803 (25 ng/mL) or vehicle control for 10 d. ALT-803 and vehicle control were replenished every 2 d. NK cell viability was analyzed on day 1, 2, 4, 8, and 10 post-treatment **(C, D)**. Results are presented as mean ± S.E.M. from three replicate wells. *p*-Values denote statistical significance relative to untreated cells (*T*-test). NK, natural killer.

To evaluate whether modulation of NK cell gene expression by ALT-803 affected NK cell viability, purified NK cells from two healthy donors were cultured with ALT-803 (25 ng/mL) or vehicle control for 6 or 10 d. As shown in Figure 5A and B, NK cells exposed to ALT-803 for 6 d showed a significant increase of viability only on day 2 and 3 in both donors compared to untreated cells (Donor 9: day 2, *p* = 0.011, day 3, *p* = 0.006; Donor 10: day 2, *p* = 0.013, day 3, *p* = 0.008). Conversely, replenishment of ALT-803 every 2 d not only resulted in a significant increase of viability starting from day 2 but also in a prolongation of viability compared to untreated cells in both donors (Fig. 5C, D).

## Discussion

The antitumor activity of some mAbs used as cancer therapeutics is exerted mainly through ADCC, a mechanism mediated by the binding between the constant region (fragment crystallizable, Fc) of mAbs and the Fc gamma receptor IIIa (FcƴRIIIa, CD16) expressed on macrophages and NK cells.^41–47^ The authors have previously reported the preclinical antitumor activity of NEO-201, a tumor antigen-targeting mAb derived from the Hollinshead allogeneic colorectal cancer vaccine platform.^36–40^ In this study, they evaluated the effects of ALT-803, an IL-15 superagonist complex consisting of an IL-15 variant (IL-15N72D) bound to an IL-15 receptor α/IgG1 Fc fusion protein, on the capacity of human NK cells to perform ADCC mediated by NEO-201.

NEO-201 is an IgG1 mAb that exhibits ADCC and CDC activity *in vitro* and attenuation of tumor growth in xenograft models.^40^ The authors demonstrated that ALT-803 significantly enhanced the ADCC mediated by NEO-201 against the highest NEO-201-positive carcinoma cell line (CFPAC-1) in a dose-dependent manner, compared with the vehicle control at both E:T ratios (Fig. 1). They also demonstrated that ALT-803, at the highest dose (25 ng/mL), significantly enhanced NEO-201-mediated ADCC at both E:T ratios in all human carcinoma cell lines, compared to untreated cells (Fig. 2), and that ADCC mediated by NEO-201 enhanced by ALT-803 is dependent on CD16 engagement (Fig. 4).

Moreover, it is interesting to note that ALT-803 retained the ability to enhance NEO-201-mediated ADCC at NEO-201 doses as low as 0.1 μg/mL. The authors also observed that NEO-201 ADCC activity at the lowest dose in presence of ALT-803 was higher than ADCC activity achieved by NEO-201 alone at the highest dose (Fig. 3), suggesting that ALT-803 could decrease the dose of NEO-201 required to achieve its clinical efficacy if used in a combined therapy.

To further investigate the mechanism by which ALT-803 enhances the ADCC mediated by NEO-201, the authors performed flow cytometry analysis on human NK cells after exposure to ALT-803. As shown in Table 2, the authors demonstrated that ALT-803 modulates the phenotype of human NK cells toward a more active cytotoxic function, increasing the expression of NK markers involved in NK cell activation and cytotoxicity (TIM-3, NKG2D, granzyme B, and CD107a).

In another study, it has been shown that short-term ALT-803 stimulation significantly increased granzyme B and perforin expression, as well as IFN-γ production in human NK cells, resulting in increased ADCC directed by an anti-CD20 mAb against B cell lymphoma cells.^19^

Similar results were achieved in other two studies, in which ALT-803 was found to enhance the function of NK cells against several ovarian cancer cell lines, multiple myeloma, and leukemia target cells with significant increases of CD107a, IFN-γ, and TNF-α expression.^24,48^

The cytokine IL-15 plays a crucial role in the immune system by affecting NK cell development, proliferation, cytotoxicity, and cytokine production.^15^

In this regard, the use of IL-15 superagonist complex (ALT-803) to enhance the NK antitumor activity has been proven to be more efficient than native IL-15. Pharmacokinetic analysis conducted in mice indicated that ALT-803 has a half-life much longer than half-life of IL-15, resulting in improved stability, longer persistence in lymphoid tissues, and enhanced antitumor activity compared to native IL-15 *in vivo*.^18^

It has been also shown that a single intravenous dose of ALT-803, but not IL-15, eliminated well-established tumors and prolonged survival of mice bearing multiple myeloma,^22^ and that ALT-803 has superior antitumor activity over IL-15 in mice bearing subcutaneous B16F10 melanoma and CT26 colon carcinoma metastases.^26^ Tissue biodistribution studies in mice also revealed a much greater retention of ALT-803 in the lymphoid organs compared to IL-15, consistent with its highly potent immunostimulatory and antitumor activities *in vivo*. In addition, multidose ALT-803 administration to cynomolgus monkeys resulted in dose-dependent increases in peripheral lymphocytes, primarily NK cells, and CD8 and CD4 memory T cells.^26^

To better understand ALT-803's mechanism of action in the modulation of NK function, the authors performed gene expression analysis on the NK cells treated with ALT-803 using the NanoString Technologies nCounter Platform. As shown in Table 3, ALT-803 was able to modulate the phenotype of human NK cells through upregulation of gene expression of NK-activating receptors, antiapoptotic factors, and factors involved in the NK cytotoxicity, and through the downregulation of gene expression of NK-inhibiting receptors and factors involved in the in the NK cell exhaustion.

It is of interest to note that ALT-803 upregulated not only the gene expression of factors mainly involved in NK killing activity, such as granzyme A and B, perforin, IFN-γ, TNF-α, and TNF-β, but also of other factors involved in NK cytotoxicity, such as clusterin and IDO. Clusterin is a secreted protein that regulates the expansion and the effector function of NK cells through the enhancement of IFN-γ production by NK cells.^54^ IDO has been found to play an important role in antitumor immunity by regulating cytotoxic activity of NK cells. Kai et al. showed that the cytotoxic activity of mouse NK cells was reduced by IDO inhibition *in vitro* and *in vivo.*^58^ The increased IDO gene expression the authors observed in human NK cells after exposure to ALT-803 could suggest an alternative mechanism by which ALT-803 enhances the cytotoxic activity of NK cells.^7^

In addition, the authors have demonstrated that ALT-803 stimulation prolongs NK cell viability (Fig. 5). This phenomenon could be, in part, due to the ability of ALT-803 to upregulate gene expression of factors involved in NK survival, such as the antiapoptotic proteins Bcl-2 and DUSP-4 (Table 3). The antiapoptotic protein bcl-2 has been found to be an activation- or proliferation-associated marker of normal NK cells, which can be induced by IL-2.^71^ DUSP-4, also called MKP-2 (mitogen-activated protein kinase 2), is expressed by NK cells. This kinase exerts antiapoptotic effects and plays a role in protecting cells against genotoxic stress-induced apoptosis.^72,73^

The use of the cytokine IL-15 to boost the immune system against cancer cells has significant potential in cancer immunotherapy. A recent clinical trial employing *Escherichia coli*-produced rhIL-15, administered to patients with metastatic malignant melanoma or metastatic renal cell cancer, demonstrated safety of IL-15 treatment of patients with metastatic malignancy. IL-15 administration markedly affected the function and proliferation NK cells in these cancer patients.^17^

Promising preclinical data on the use of ALT-803 as an anticancer drug have spurred interest in using it in the clinic. Several clinical trials are ongoing to evaluate the safety and efficacy of ALT-803 alone or in combination with conventional cancer treatments.

For example, a phase I clinical trial will investigate the safety and immunogenicity, immunomodulatory properties, and clinical benefits of treatment with weekly doses of ALT-803 in patients with surgically incurable advanced solid tumors, including melanoma and renal cell, nonsmall cell lung and squamous cell, and head and neck cancer.^28^ Recruiting clinical trials will also evaluate the efficacy of ALT-803 as a therapy for the treatment of ovarian cancer, acute myelogenous leukemia, myelodysplastic syndrome, and relapsed or refractory multiple myeloma.^29–31^ ALT-803 will also be administered in combination with pembrolizumab or nivolumab in patients with advanced or metastatic nonsmall cell lung cancer, who have failed treatment with PD-1 checkpoint inhibitor therapy^32^; with rituximab in patients with relapse/refractory indolent B cell non-Hodgkin lymphoma^33^; with Intravesical BCG in patients with BCG-unresponsive high-grade nonmuscle invasive bladder cancer^34^; and with ETBX-011 (Ad5 [E1-, E2b-]-CEA(6D)) vaccine in subjects having CEA-expressing cancer.^35^

## Conclusions

This investigation has demonstrated that ALT-803 significantly enhanced the ADCC activity mediated by NEO-201 against NEO-201-positive human carcinoma cells. The enhancement of NEO-201-mediated ADCC may be due to the ability of ALT-803 to increase the expression of TIM3, NKG2D, granzyme B, and CD107a on CD56^+^/CD16^+^ NK cells, as well as to its capacity to upregulate gene expression of NK-activating receptors, factors involved in the NK cytotoxicity, and antiapoptotic factors.

The improved stability and enhanced antitumor activity of ALT-803 compared to native IL-15 *in vivo* offer a good chance to use it in combination with NEO-201 in clinic. NEO-201 pharmacokinetics evaluation in nonhuman primates showed that NEO-201 half-life was 167 or 170 h at the 20 or 49 mg/kg dose, respectively.^40^

The long permanence in the bloodstream of both drugs suggest that ALT-803 could enhance the NEO-201 antitumor activity *in vivo* in humans, supporting rationale for the clinical development of the combination therapy using NEO-201 and ALT-803 to treat patients with a broad variety of carcinomas.
